# Supplementary figures and images for: Candida albicans commensalism in the oral mucosa is favoured by limited virulence and metabolic adaptation
Source: PLoS Pathog. 2022 Apr 11;18(4):e1010012. doi: 10.1371/journal.ppat.1010012 (PMC9041809; doi:10.1371/journal.ppat.1010012)

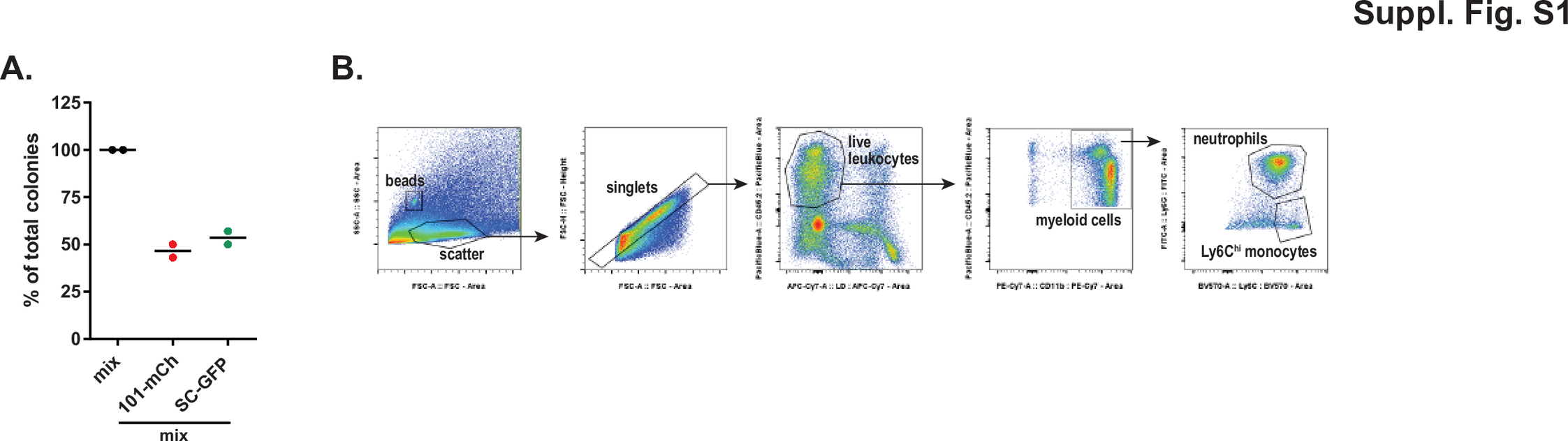

Supplement: S1 Fig — Isolate 101 persists in the oral mucosa even in presence of SC5314-induced inflammation. A. The proportion of each isolate 101-mCherry and SC5314-GFP in the infection inoculum ‘mix’ was analysed prior to infection. B. Gating strategy for quantification of neutrophils and inflammatory monocytes in the infected tongue on day 1 post-infection. (TIF) [file ppat.1010012.s001.tif]

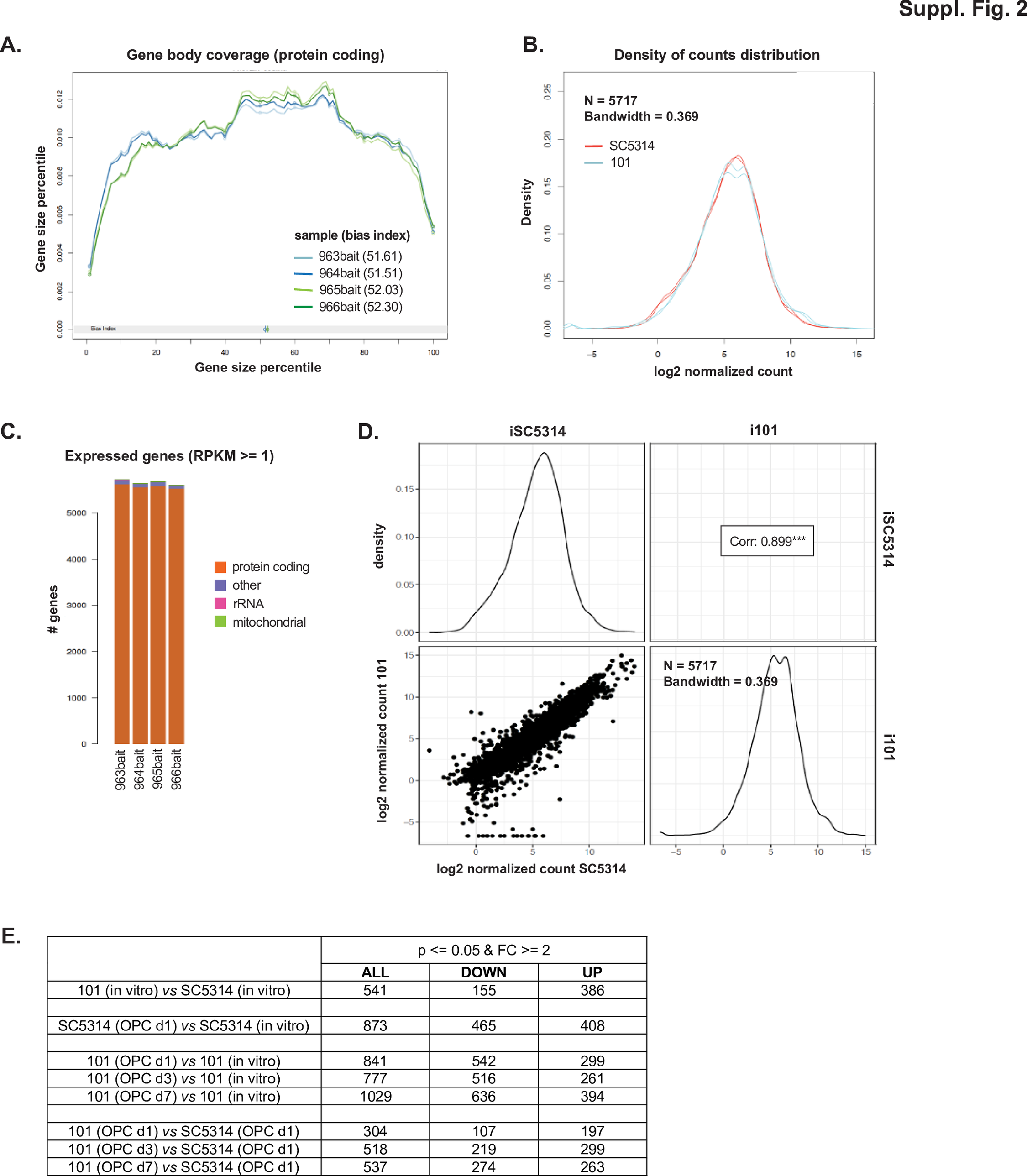

Supplement: S2 Fig — Read alignments parameters in RNAseq data analysis for SC5314 and 101. A. Gene body coverage represents the distribution of reads on each gene for each sample of strain SC5314 (963bait and 964bait) and 101 (965bait and 966bait) tested. B. Count distribution represents the distribution of gene counts between the four samples. C. Number of expressed genes (RPKM ≥1) in each of the categories protein coding, rRNA, mitochondrial and other. D. Correlation of gene expression by tested samples of isolates SC5314 and 101. E. Numbers of globally up- and downregulated genes between SC5314 and 101 and between the different conditions tested. (TIF) [file ppat.1010012.s002.tif]

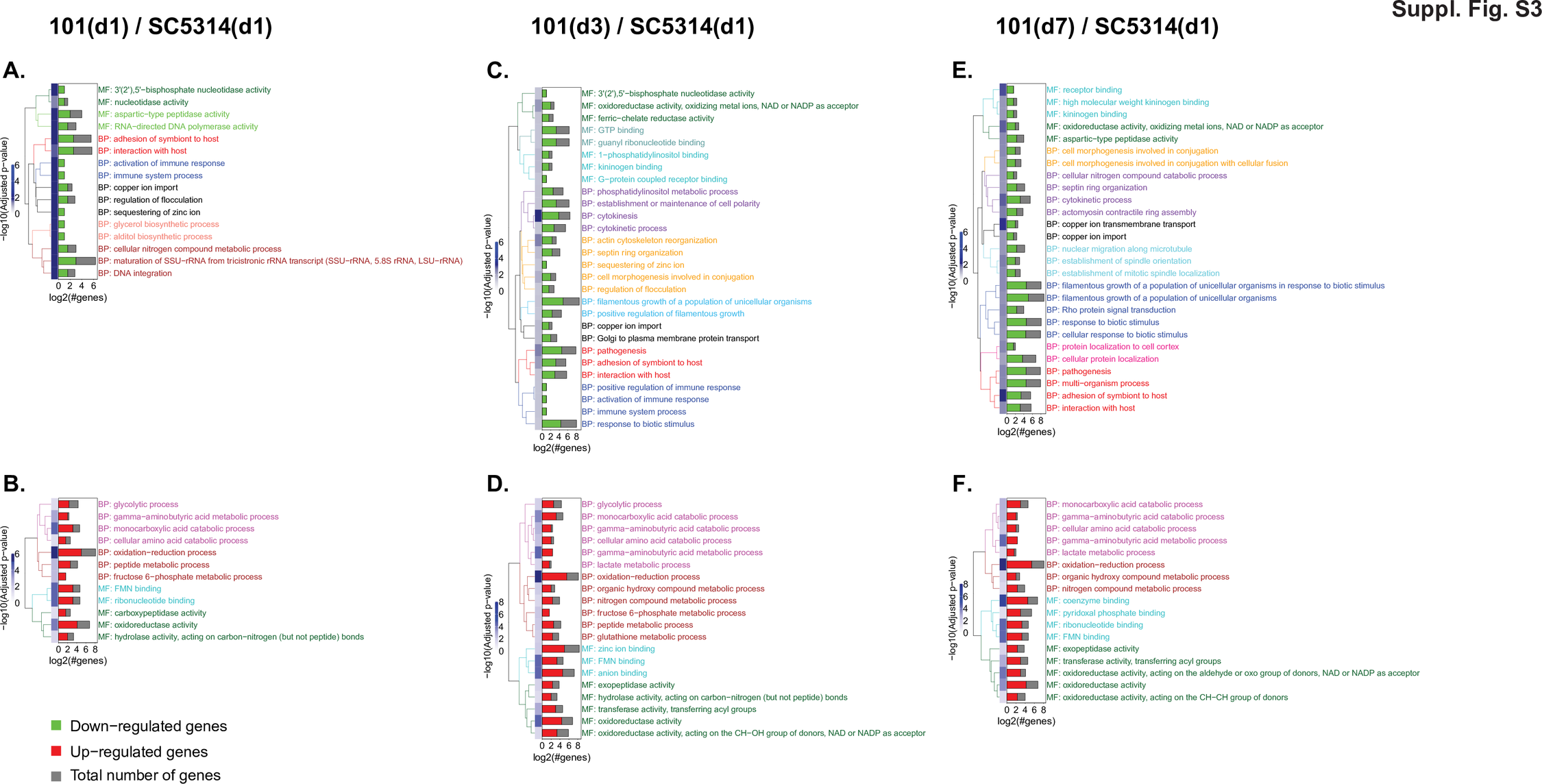

Supplement: S3 Fig — GO terms associated with differentially regulated C. albicans genes in the infected tongue. A-F. Enriched GO terms (adjusted p-value < 0.05) on Biological Process and Molecular Function associated with down-regulated (A, C, E) and up-regulated genes (B, D, F) between isolate 101 on day 1 (A, B), day 3 (C, D) or day 7 (E, F) and SC5314 on day 1 post-infection. GO terms are arranged by hierarchical clustering using Wang-measure semantic similarity. The enrichment adjusted p-value (-log10) is indicated. The horizontal bars represent the number (log2) of differentially regulated genes against the total number of genes associated with each GO term. (TIF) [file ppat.1010012.s003.tif]

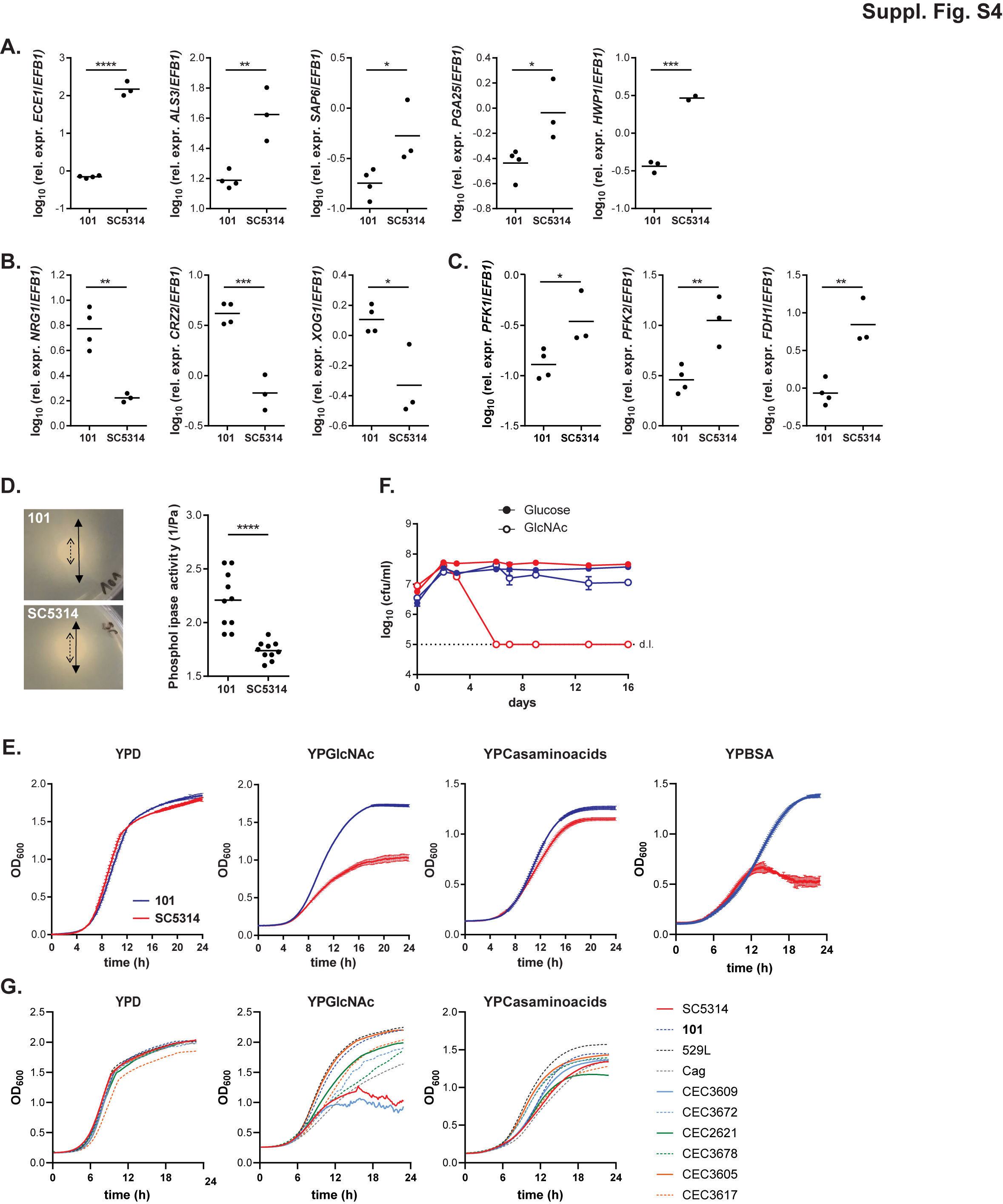

Supplement: S4 Fig — Confirmation of differentially regulated genes and validation of the altered metabolic signature of isolate 101. A-C. Isolate 101 and SC5314 were exposed to monolayers of TR146 keratinocytes for 24 hours and expression of the indicated fungal genes was assessed by RT-qPCR. Selected virulence and morphogenesis genes downregulated in isolate 101 are shown in A, selected transcription factors and cell wall genes upregulated in isolate 101 are shown in B, and differentially regulated genes associated with metabolism are shown in C. D. Phospholipase activity was assessed after incubation of isolate 101 and SC5314 on Prices’ original egg yolk agar for 4 days at 37°C and 5% CO2. Representative images are shown on the left. The percentage of phospholipase activity (right) was calculated from the ratio of the diameter of the colony (dotted arrow) relative to the diameter of the precipitation zone formed around the colony (solid arrow). E. Growth curves for isolates 101 and SC5314 cultured in YPD, YPGlcNAc, YPCasaminoacids or YPBSA medium, respectively. Data are the mean±SD of at least two technical replicates of each sample and representative of at least 3 independent experiments. F. Isolates 101 and SC5314 were cultured in H2O containing Glucose (filled symbols) or GlcNAc (open symbols) and the number of viable fungal cells was quantified at the indicated time points to assess GlcNAc-induced cell death. Each symbol is the mean±SD of duplicate measurements. Data are representative of 3 independent experiments. G. Growth curves for the indicated high-damage inducing (solid line) and low-damage inducing (dotted line) C. albicans isolates cultured in YPD, YPGlcNAc or YPCasaminoacids medium, respectively. Data are the mean±SD of two technical replicates of each sample and representative of 2 independent experiments. (TIF) [file ppat.1010012.s004.tif]

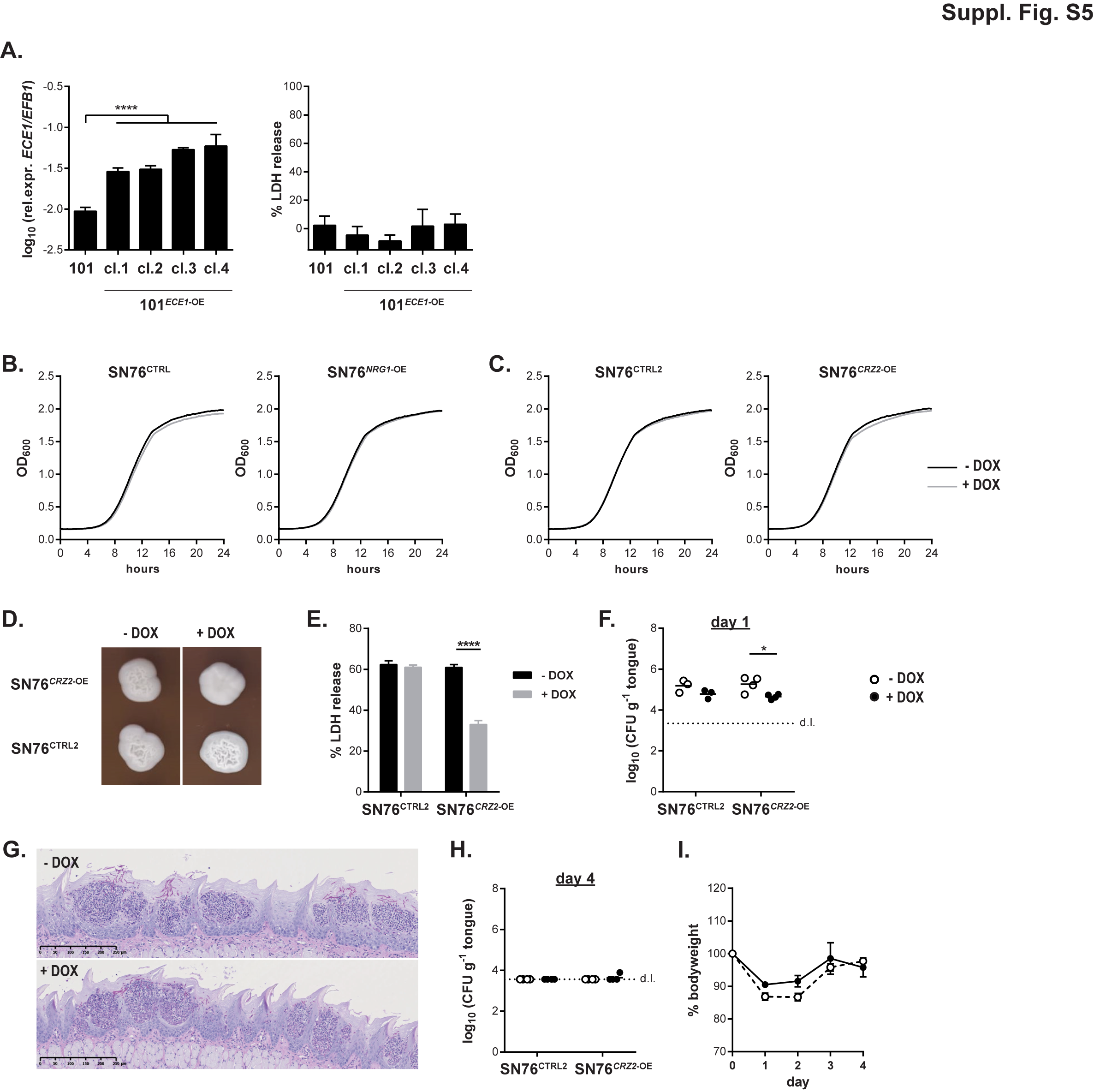

Supplement: S5 Fig — A. Overexpression of the ECE1 gene (from SC5314 under the TDH3 promoter in isolate 101. ECE1 expression levels (left panel) and LDH release (right panel) were assessed in four different clones in comparison to the parental isolate 101 after exposure to monolayers of TR146 keratinocyte for 24 hours. B.-C. Growth curves of SN76NRG1-OE (B) or SC5314CRZ2-OE (C) and corresponding controls in presence and absence of Dox. Each line is the mean of 4 samples per condition. D. Morphology of SC5314CRZ2-OE and SC5314CTRL on YPD agar with or without Dox for 2 days. E. Monolayers of TR146 keratinocyte were infected with SC5314CRZ2-OE or SC5314CTRL in presence or absence of Dox and epithelial cell damage was assessed after 24 hours of infection by LDH release assay. Bars are the mean+SEM of 8 samples per condition pooled from 2 independent experiments. F.-I. C57BL/6 WT mice were infected sublingually with SC5314CRZ2-OE or SC5314CTRL and treated or not with Dox. Fungal burden was assessed after 1 day (F) or 4 days of infection (H). Tongue sections were stained with PAS on day 1 post-infection (G). Weight loss and re-gain relative to the pre-infection weight is shown in (I). In F and H, each symbol represents one animal, in I each symbol is the mean±SD of 4 animals. (TIF) [file ppat.1010012.s005.tif]

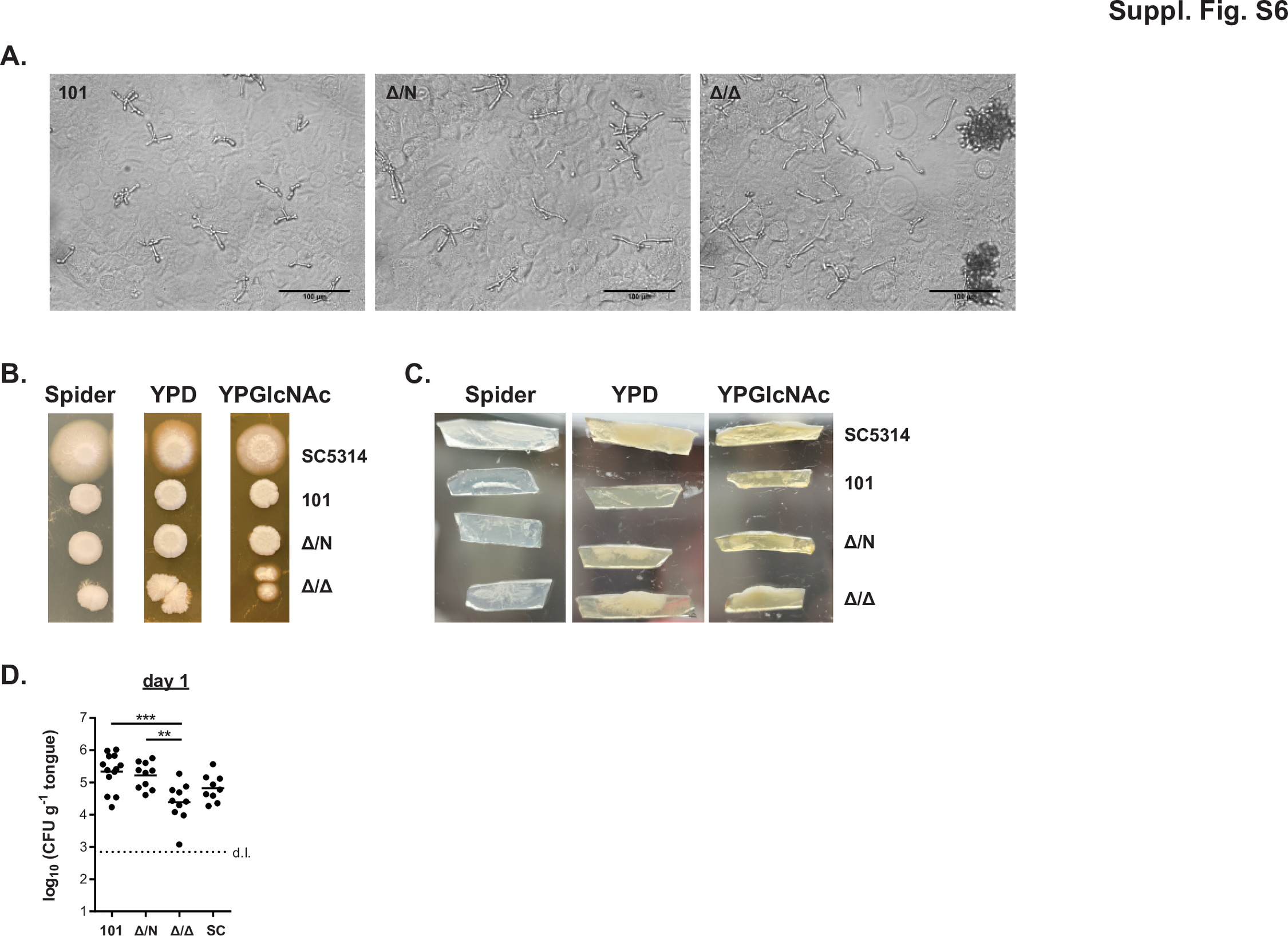

Supplement: S6 Fig — Reduced expression of NRG1 in isolate 101 increases its pathogenicity. A. Representative images used for measuring hyphae length shown in Fig 6C for isolates 101nrg1Δ/NRG1, 101nrg1Δ/Δ, the parental isolate 101 on monolayers of TR146 keratinocytes in F12 medium. B-C. Growth of isolates 101nrg1Δ/NRG1, 101nrg1Δ/Δ, the parental isolate 101 and SC5314 on Spider agar (left), YPD agar (middle) and YPGlcNAc agar (right) for 8 days at 30°C. Colonies were imaged from top (B) or from the side after cutting the agar from top to bottom (C). D. Fungal burden in the tongue of C57BL/6 WT mice that were infected sublingually for 1 day with isolate 101nrg1Δ/Δ in addition to 101nrg1Δ/NRG1, the parental isolate 101 and SC5314 as in Fig 6E. (TIF) [file ppat.1010012.s006.tif]

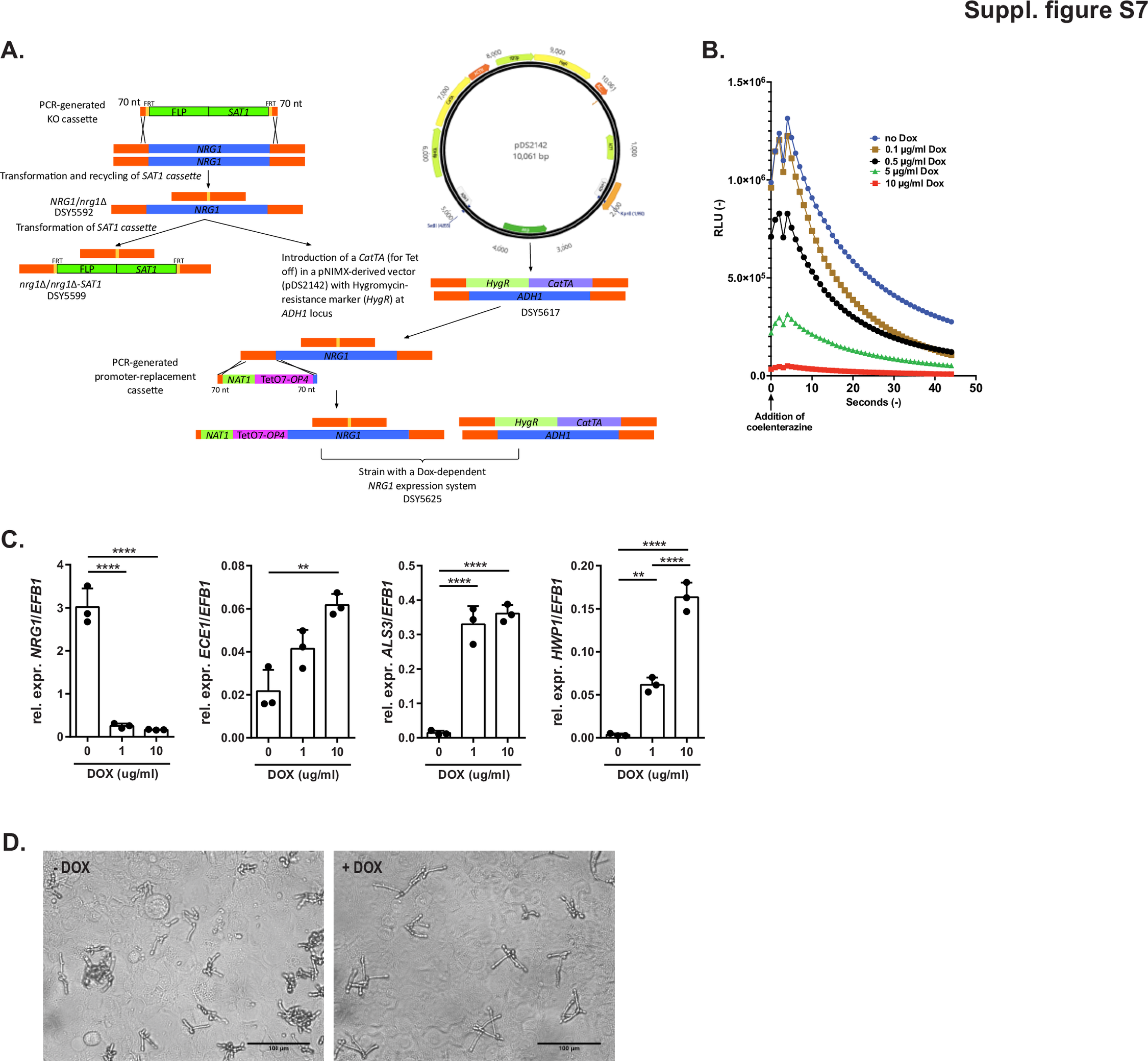

Supplement: S7 Fig — Suppression of NRG1 expression in isolate 101 via a TET-off strategy drives fungal pathogenicity. A. Strategy used for generating isolate 101nrg1Δ/pTET-NRG1 using a Dox shut-off system (pTet-off). Deletion of NRG1 was performed with a PCR-generated KO cassette. Experimental details are given in the Methods section. FLP, Flippase; RFT, flippase recognition target; NAT1 and SAT1, nourseothricin resistance genes; CatTA, C. albicans tetracycline transactivator; HygR, hygromycin resistance. B. Functionality of the Tet-off system was tested with a luciferase reporter system. Median values of duplicate measurements for each condition are shown. RLU, relative luminescence units. C. Monolayers of TR146 keratinocytes were infected with isolates 101nrg1Δ/pTET-NRG1 in presence of 0, 1 or 10 μg/ml Dox for 24 hours. Expression of the indicated fungal genes was assessed by RT-qPCR. Bars are the mean+SD of 3 samples per condition from a single experiment. D. Representative images used for measuring hyphae length shown in Fig 7D for isolate 101nrg1Δ/pTET-NRG1 with or without 10 μg/ml Dox on monolayers of TR146 keratinocytes in F12 medium. (TIF) [file ppat.1010012.s007.tif]
