# Supplementary material for: Candida albicans commensalism in the oral mucosa is favoured by limited virulence and metabolic adaptation
Source: PLoS Pathog. 2022 Apr 11;18(4):e1010012. doi: 10.1371/journal.ppat.1010012 (PMC9041809; doi:10.1371/journal.ppat.1010012)
Supplement: S1 Table — Alignment metrics of two duplicated control RNA-seq samples of SC5314 and 101. (DOCX) [file ppat.1010012.s008.docx]

*Supplementary Table S1. Alignment metrics of two duplicated control RNA-seq samples of SC5314 and 101*

| **Metric** | **963bait** | **964bait** | **965bait** | **966bait** |
| --- | --- | --- | --- | --- |
| Total_reads | 57256170 | 53140585 | 52523170 | 57209232 |
| PF_reads | 57256170 | 53140585 | 52523170 | 57209232 |
| PF_reads_aligned | 56589845 | 52473632 | 51534374 | 56065563 |
| Pct_PF_reads_aligned | 98.84 | 98.74 | 98.12 | 98.00 |
| PF_aligned_bases | 5640845923 | 5217031794 | 5120355346 | 5582997636 |
| Mean_read_length | 100.08 | 99.85 | 100.00 | 100.19 |
| Reads_aligned_in_pairs | 0 | 0 | 0 | 0 |
| Pct_reads_aligned_in_pairs | 0 | 0 | 0 | 0 |
| Strand_balance | 0.48 | 0.48 | 0.47 | 0.48 |
| Unpaired_read_duplicates | 54645694 | 50449999 | 50119344 | 54509132 |
| Read_pair_duplicates | 0 | 0 | 0 | 0 |
| Percent_duplication | 96.56 | 96.14 | 97.25 | 97.22 |
| Uniquely_aligned | 54829321 | 50830133 | 49152181 | 53465285 |
| Multiple_alignment | 1760524 | 1643499 | 2382193 | 2600278 |
| Read_aligned_to_ref_exons | 52695641 | 48990572 | 47704147 | 51753699 |
| No_feature | 2087964 | 1796337 | 1390847 | 1650562 |
| Ambiguous | 45716 | 43224 | 57187 | 61024 |
| Too_low_aqual | 0 | 0 | 0 | 0 |
| Not_aligned | 0 | 0 | 0 | 0 |
| Alignment_not_unique | 3860571 | 3631709 | 5169509 | 5636098 |
| Reads_aligned _to_rRNA | 1988202 | 1818577 | 1462204 | 553094 |
| Reads_aligned _to_rRNA_% | 3.47 | 3.42 | 2.78 | 0.97 |
| Reads_aligned_to_mitochondria | 25434 | 23882 | 23359 | 12879 |
| Reads_aligned_to_mitochondria_% | 0.04 | 0.04 | 0.04 | 0.02 |
| bias_index_50 | 52.62 | 52.51 | 53.02 | 53.26 |
| 5over3_cov_ratio | 0.91 | 0.92 | 0.88 | 0.85 |
