# Supplementary material for: Candida albicans commensalism in the oral mucosa is favoured by limited virulence and metabolic adaptation
Source: PLoS Pathog. 2022 Apr 11;18(4):e1010012. doi: 10.1371/journal.ppat.1010012 (PMC9041809; doi:10.1371/journal.ppat.1010012)
Supplement: Attachment — Submitted filename: point-to-point reply_final.docx [file ppat.1010012.s010.docx]

**POINT-TO-POINT REPLY**

**Reviewer #1:**

The study by Lemberg et al. focuses on the oral colonization and pathogenicity determinants of one of the major fungal pathogen of humans, Candida albicans. The work compares side by side the colonization and virulence characteristics of C. albicans isolate strain 101 and the typical reference control SC5314. SC5314, originally a bloodstream isolate that has been lab propagated for many years, is known for its robust virulence phenotypes like avid filamentation and tissue damage. In this study Lemberg et al. once again show that the damaging potential of SC5314 exceeds that of other strains (101) and provide an evidence for it - differences in NRG1, a known hyphal repressor. Alteration of NRG1 levels was sufficient to change the invasiveness in the oral model. Importantly, the study also reveals significant differences in utilization of nutrients by the two strains in support to increasing amount of evidence that C. albicans metabolism and virulence are tightly connected.

Major issues:

The provided data and the following discussion does not address the following:
1. Is 101 or SC5314 the outlier here? The two strains originate from two different niches (oral vs. blood (with unknown commensal origin)) and it will be important to test another oral isolate shown to cause SC5314-like damage. Will the filamentation and metabolic repertoire of other strain(s) be more 101 or SC5314-like?

In our previous publication by Schönherr et al. (doi: 10.1038/mi.2017.2), we tested the behaviour of a number of different isolates for their capacity to induce damage in oral epithelial cells and for their behaviour in the oral mucosa of C57Bl/6 mice, i.e. under the same conditions as in the current study. Among the examined isolates was the isolate CEC3605, a commensal isolate from a healthy carrier, which induces high epithelial cell damage and causes a strong inflammatory response in the oral mucosa of mice, similar to strain SC5314 (i.e. an isolate meeting the criteria quested by the reviewer). In the same paper we also showed that among a collection of 76 isolates, the majority was low-damage inducing and only about a quarter of all tested isolates was high damage inducing, whereby the damage inducing capacity does not correlate with the origin from mucosal surfaces or from blood cultures (see figure 5E-F in Schönherr et al., doi: 10.1038/mi.2017.2). We have in the meantime expanded the analysis of epithelial damage induction with >200 isolates and confirmed our original conclusions. Therefore, strain SC5314 rather than isolate 101, appears to be the outlier regarding its high virulence.

When looking at a broader set of fungal characteristics, beyond epithelial cell damage induction, it becomes clear that every isolate is unique in how it combines different phenotypic and functional features. While in the OPC model, high damage induction in epithelial cells inversely correlates with overall enhanced persistence in the murine oral cavity (shown for 7 isolates in Schönherr et al. (doi: 10.1038/mi.2017.2) and confirmed for additional high- and low-damage isolates in the meantime (unpublished results)), every isolate is somewhat different. The notion that individual isolates display multiple phenotypic differences has also been highlighted by a recent study from the Ene, Hohl and Koh labs, which has been published since the initial submission of our manuscript (doi: 10.1128/mBio.02878-21) and is now cited in our manuscript (line 514ff). Therefore, whether two strains are classified as similar or not depends on the feature (or the combination of features) that is/are analysed. As such, *C. albicans* isolates also differ in their metabolic profile as assessed here as their preference to grow in GlcNAc- or casamino acid-containing medium. We have included a new figure showing the growth of several low- and high-damage inducing isolates (including isolate CEC3605 mentioned above), which shows no association between the isolates’ growth under the tested conditions and their damage-inducing capacity (new **Figure S4G**, accompanying text in the results section (lines 282ff), the methods (Table 1), the discussion (line 506ff) and the supplementary figure legends (line 1353ff)). Thus, neither strain SC5314 nor isolate 101 represents the rule or the exception, and other isolates are either SC5314-like, 101-like or anywhere in between, depending on the feature being looked at.

2. What is the reason for this difference in NRG1-coordinated effects between the strains? A difference in NRG1 regulation? Can and how this connects to the metabolic adaptation? As NRG1 is relatively well studied it is worthy to take a closer look at the promoter region and provide some more direct evidence for the observed phenotypic differences.

We thank the reviewer for raising this important question, which addresses the pronounced differential expression of *NRG1* between isolate 101 and SC5314. Sequence differences between the two strains are numerous. However, we did not identify any significant differences in the promoter region of *NRG1* between the two strains that could explain the differential expression of *NRG1*. Among the observed sequence differences between the two strains, we found an approximately 200 base pair duplication of the C-terminal end of the gene encoding Brg1, a known regulator of *NRG1* expression and thus a likely candidate for the *NRG1*-dependent differences between isolates SC5314 and 101. This duplication, which is present in one of the two alleles, resulted in a frameshift and truncation of one gene copy. To correct this defect in isolate 101, we generated gene copy replacement mutants and carried out several functional experiments in vitro and *in vivo*. However, resolving of a single genetic difference between SC5314 and 101 was not enough for modulating *NRG1* expression or for rendering isolate 101 more virulent. Despite these negative results, we still cannot exclude that *BRG1* truncation may contribute to the phenotype of isolate 101 in combination with other genetic determinants. In this respect, heterozygous deletion of *BRG1* was recently found to alter infectivity of SC5314 in the OPC model using immunosuppressed mice, but only if at least one copy of *EFG1* was deleted simultaneously, indicating that Brg1 acts not alone to regulate *NRG1*-restricted pathogenicity in the oral mucosa (doi: https://doi.org/10.1101/2021.05.24.445409). Exploring additional candidate genetic traits in isolate 101, and combinations thereof, that might be responsible for high *NRG1* expression and the commensal phenotype goes beyond the scope of this study. Given the negative results obtained with the *BRG1* gene replacement mutants we decided not to include the results in the manuscript (data can be provided upon request). Instead, we expanded the text in the discussion section (lines 438ff).

Regarding the question whether high *NRG1* expression connects to the metabolic adaption in isolate 101, we assessed growth of the *NRG1* deletion mutant 101*^nrg1Δ/^*^NRG1^ under the different growth conditions, which we used in supplementary Figure S4E. Results from these experiments revealed that reduction *NRG1* expression levels by deletion of one allele did not markedly alter the nutrient preferences of isolate 101, at least under the tested conditions (see Figure A below). Of note, due to the clumping phenotype of the homozygous mutant 101*^nrg1Δ/Δ^*, growth curve experiments did not provide meaningful results. A link between *NRG1* and *C. albicans* metabolism is therefore not obvious from these data, although we cannot exclude that deletion of only one allele is insufficient for revealing an effect. Likely, other differences between SC5314 and 101 than the expression levels of *NRG1*, are responsible for the strain-specific metabolic profile, while *NRG1* is linked to virulence and has a remarkable effect on the fate of isolate 101 *in vivo* in the oral mucosa.

***Figure A:*** *Growth curves for isolates SC5314 (solid red line), 101 (solid blue line) and the mutant 101^nrg1Δ/NRG1^ (dotted blue line) cultured in YPD, YPGlcNAc, YPCasamino acids or YPBSA medium, respectively. Data are the mean±SD of two technical replicates of each sample.*

3. Previous reports show that nitrogen utilization by Candida is important in oral infections. The data also suggests that 101 has different efficiency of utilizing nitrogen sources. Testing the repertoire and efficiency of utilization of simple (amino acids/peptides) and more complex (oligopeptides/proteins) nitrogen sources is relatively simple, but would enhance the understanding about fungal persistence in this host niche.

We thank the reviewer for his/her valuable suggestion. We generated new data on isolate 101’s efficiency to utilize simple and more complex nitrogen sources and found that it has an advantage over strain SC5314 not only to use casamino acids but also protein (BSA) as a nitrogen source. The results from these experiments are included in a new Figure (**new Supplementary FigureS4E**). The text has been adapted accordingly (lines 272f and 1346ff).

Minor Issues:

1. The manuscript is interesting, but at the end remains unclear what it will take for such persistent colonization and low invasiness in the oral cavity to transition into invasive and symptom causing infection. The data shows that this switch is NRG1 coordinated. So, within the oral cavity, when and how mechanistically this switch to the more virulent form will occur? Such discussion (and addressing my major issue #2) will significantly enhance the manuscript.

While the question raised by the reviewer is of significant relevance, we would like to emphasize that our study focusses on the natural intraspecies variation of *C. albicans* and what influences colonization of the oral niche, and not on the switch from a commensal to a pathogenic state. We focus on the prototypical commensal isolates 101 that is increasingly being used by the community as an avirulent representative of the species of *C. albicans*, as it becomes more and more clear that SC5314 is not representative of the entire species. Our results highlight that commensalism of isolate 101 in this niche is determined by a combination of several features, including filamentation, damage induction and metabolic adaptation, and that at least some of these features are coordinated by the transcriptional regulator *NRG1*.

How a particular commensal isolate such as isolate 101 may become more virulent and what conditions favour such a switch is a different question. As we have learned from recent studies by Kyla Ost et al. (doi: 10.1038/s41586-021-03722-w) and Itai Doron et al. (doi: 10.1038/s41564-021-00983-z), this process can be host-driven. Of interest, in *Rag1-/-* deficient mice colonized with *C. albicans* *NRG1* expression is downregulated along with the induction of numerous *C. albicans* virulence genes. Assessing whether and under which conditions isolate 101 switches from a commensal into a more pathogenic form in the oral cavity and how *NRG1* may be involved in this process is part of an independent project in the LeibundGut-lab and will be the topic of a separate manuscript.

2. The manuscript needs to be checked throughout for styling issues (spacing, etc.), some grammatically incorrect/overly edited sentences and most importantly - please check if all Materials and Methods are in place as I could not locate some information (like medium composition).

We have carefully revised the manuscript. In particular, we have edited the methods section and added more details regarding medium composition (see e.g. lines 646ff and 663ff).

3. I found the introduction relatively difficult to go through as to me the information was not presented in a logical order. Perhaps this is just a personal preference, but I think the flow can be improved. For instance, some of the details in the introduction can be moved to the discussion, which on the other nahd can be expanded. Importantly, a wonderful study by the Jacobsen lab recently published data that metabolic advantage and not filamentation is important for gut pathogenicity. This observation should be discussed.

Taking the advice of the reviewer we have revised the introduction to make it easier to follow. The discussion has also been revised and expanded.

The paper from the Jacobsen lab (Dunker et al.,), which was already cited in the original version of our manuscript, is now discussed more extensively in the context of the role of metabolic adaptation for pathogenicity (lines 496ff)

**Reviewer #2:**

This is a well-written and interesting manuscript examining the capacity of a clinical strain of C. albicans to colonize murine oral mucosal. Strengths include the genetic modulation on nrg1 in multiple systems and showing a link of the suppressor of filamentation to the colonization phenotype. Multiple other virulence traits are considered. Targeted and nontargeted approaches are complementary. A limitation is the use of a single clinical strain as a comparison to SC5314. Some of the legends should be clarified.

We would like to thank the reviewer for his/her positive evaluation of the manuscript and for highlighting the strength of our complementary approaches, including the multiple genetic approaches, to demonstrate a link between the transcriptional repressor Nrg1 and the colonization phenotype.

In reply to the limitation of using a single strain, which was also raised by reviewers #1, we have performed new experiments and expanded the discussion to highlight that isolate 101 is not an outlier, neither with respect to its low damage-inducing capacity, its colonization of the oral mucosa, nor to its metabolic profile. See our more extensive answer to reviewer #1 above, the **new Figure S4G**, line 282ff, lines 506ff and lines 1353ff in the manuscript.

Clarify the data for Figure 2D-H legend. Were experiments performed on multiple days? The way the legend reads, the data included replicate wells from a single experiment.

Data from reconstituted epidermis are from a single experiment. This has been clarified in the legend (line 957f).

Figure 4B-C would benefit from additional labeling or explanation. It is not clear what the different colors mean.

The figure panels have been modified. Each of the three overlapping coloured areas represents a time point of analysis, which are now labelled. The legend has been adapted accordingly (line 981).

Figure 4D: How were the genes selected for display?

The genes displayed in the heat map in Figure 4D are amongst the most strongly regulated genes highlighting some gene categories related to the GO terms that were enriched (see **Figure 4B-D** and **Supplementary Figure S3**). The full table of differentially regulated genes has been included in **new Supplementary Table S2**.

Figure 5B-C: Clarify in the legend, should this read right/left (versus top/bottom)?

We apologize for the error in the legend, which has been corrected (line 998).

Figure 6A-B. 7A-B: Were the experiments performed multiple times, or are the data from replicates of a single experiment?

The legends to Figures 6A-B and 7A-B have been expanded to indicate that the data shown are from one out of two independent experiments.

In addition, we have added more datapoints to figure 6H and adjusted the legend accordingly (lines 1020 and 1042).

C. albicans killing by neutrophils: This section was in methods, but I didn’t see the experiments in the main text.

We apologize for this error, which has been corrected.

Figure S2 legend: Were the strains compared at each time point, or were the various time point for 101 compared to SC5314 on day 1?

Because strain SC5314 is cleared from the murine tongue within a few days, it is not possibly to isolate RNA from this strain at any time point later than day 1. Therefore, the transcript profile of isolate 101 from each time point (day 1, day 3, day 7) was compared to the transcript profile of strain SC5314 on day 1. The labels in Figure S3 (please note that the original Figure S2 is now Figure S3) have been adjusted to make this point more transparent. The legend has been adapted accordingly (lines 1327f).

Figure S5 legend: For A, what media is used?

Figure S6 shows representative images of filamentation of *NRG1* deletion mutants, which was assessed on monolayers of TR146 keratinocytes, as described in the methods section (line 624ff and 667). The legend of Figure S6A (please note that the original Figure S5 is now Figure S6) has been adjusted to provide more clarity (lines 1380ff). During the revision, we realized that in the legend to Figure S6, the order of the panels was mixed up. This has also been corrected (lines 1385f).

Figure S6C: Are the data individual experiments of replicates?

The titration of DOX was done once. This has now been clarified in the legend to Figure S7 (Please note that the original Figure S6 is now Figure S7; see lines 1403f).

Minor: Legends should be clarified as suggested above.

Thank you for the critical reading. All legends have been adjusted as suggested.

**Reviewer #3:**

In this study by Lemberg and colleagues, the authors seek to gain deeper understanding of the fungal determinants of commensal establishment of Candida albicans in the oral epithelium. Using the previously described oral isolate 101, they characterize its biology and interactions with a mouse model host, in comparison to the pathogenic SC5314 isolate. Compared to SC5314, isolate 101 exhibited shorter filaments in multiple experimental models. Additionally, its expression of virulence-associated transcripts was decreased compared to SC5314, which correlated with less induction of host immunity in the mouse oral epithelium. The authors also found that isolate 101 had increased expression of metabolism-related transcripts. Overall, the manuscript includes a body of work, but a significant portion of the data supports previously understood phenomena, rather than generate novel understanding of the mechanisms supporting 101 persistence.

Major issues:

1. The authors focus on the Candida transcription factor NRG1, whose roles are fairly well understood. Although the authors examine transcripts that are associated with Nrg1 regulation, the function of any of these differentially genes on virulence in the 101 strain is not explored.

Nrg1 represses multiple virulence genes of *C. albicans*. In the 101 mutants with reduced or abolished expression of *NRG1* we demonstrate de-repression of several well-known *NRG1* target genes such as *HWP1*, *ALS3* and *ECE1* (see Figures 6AB, 7AB). They are well known for their association with filamentation and virulence, and they have been broadly studied, also in *in vivo* models. As such, mutants of *ECE1*, *ALS3* and *HWP1* (on the SC5314 background) have been tested in the OPC model (using immunosuppressed mice) where all mutants showed reduced virulence & infectivity (PMID 27027296 for *ECE1*; PMID: 22544909 for *ALS3* and *HWP1*). Generating deletion mutants of any of these Nrg1-repressed genes in isolate 101 is not meaningful, given the very low expression of the genes in isolate 101.

2. There is also a significant experimental/technical problem, in that the 101 transcripts were mapped to the SC5314 genome background. As this strain is quite genetically distant from SC5314, it is likely that the SNPs would alter the ability of a particular read to map to the genome. Recent work has also showed that diverse strains of C. albicans include many novel genes (529L PMID: 31221654 and CHN1 PMID: 34110235), so the transcriptome data needs to be re-analyzed against the appropriate genome reference.

We acknowledge the remark of the reviewer. We actually verified that the 101 reads would efficiently map to the baits designed on the basis of strain SC5314. We added a statement in the results section (lines 213ff), a detailed paragraph (lines 814ff) in the Methods section and included a **new Supplementary Figure S2** and a **new Supplementary Table S1** to explain this and to provide specific arguments in support of our choice of procedure.

With regards to the need of remapping the 101 reads to a 101 genome, we think this is not critical to the understanding of the manuscript. This is based on the above-mentioned argument and to the fact that we here made comparisons between the transcript profiles of SC5314 and 101 which only take into account genes common for both isolates and do not include isolate-specific genes. In addition, 101-specific read mapping would require a 101 annotated genome, which is not yet available and out of the scope of the current study.

3. From Figure 3, it is clear that the attenuation of the host immune response to isolate 101 is not responsible for its increased ability to colonize the oral epithelium, as the neutrophil response is actually highest in the mixed culture. Therefore, the focus on the immune response to the Nrg1 mutant strains seems misplaced.

Isolate 101 does not induce an inflammatory response at early time points after infection in single-strain infections, as demonstrated in a previous publication by Schönherr et al. (doi: 10.1038/mi.2017.2) and reproduced multiple times by independent investigators. This applies similarly to other low-damage-inducing isolates. For interpretation of the results in Figure 3, the spatial organization has to be considered: isolate 101 and SC5314 localize differently in the experimentally infected murine oral mucosa. SC5314 invades the stratum granulosum and stratum spinosum. In contrast, isolate 101 does not invade and is limited to stratum corneum, which is not reached by neutrophils (see Figure 1A in this manuscript and Figure 4 in Schönherr et al, doi: 10.1038/mi.2017.2), and it is therefore spared from SC5314-elicited neutrophils, in addition to displaying enhanced resistance of 101 to oxidative and other host stresses.

4. Additionally, the altered metabolism transcriptional signature phenotype is not experimentally followed up, and so the title claim is not well supported by the data.

We did experimentally follow up the metabolic signature revealed by the RNAseq data by means of functional assays, which we further expanded during the revision of our manuscript. We thereby confirmed altered nutrient preferences of the commensal isolate 101 compared to SC5314. As such, we believe that the title of our manuscript is justified.

Therefore, the manuscript is largely descriptive and does not provide significant new knowledge to the field.

We respectfully disagree with the reviewer’s notion. Although Nrg1 is a relatively well-known transcriptional repressor and regulator of filamentation in *C. albicans*, its role had been established in the high virulent lab strain SC5314 and no study so far has investigated the role of Nrg1 in a natural model of colonization. The commensal isolate 101 is increasingly being used by the community as an avirulent representative of the species of *C. albicans*, as it becomes more and more clear that SC5314 is not representative of the entire species. While the vast majority of studies assessing factors driving commensalism used the high virulent strain SC5314 and mutants thereof, we have genetically manipulated Nrg1 in isolate 101 using multiple systems to study its function in this intrinsically commensal strain and in a model of natural colonization. To our knowledge, the OPC model of infection is the only mammalian model of commensal colonization in an important niche of the fungus that does not depend on immunosuppressant, antibiotic or hormone treatment of mice. Thereby, our manuscript uses a novel approach to provide relevant mechanistic insights into what supports persistence of a prototypical commensal isolate in the mucosal niche.

Minor issues:

1. It is unclear how invasion is measured in Figure 2B, and how “percent invasive hyphae” is quantified. The authors should clarify this in the figure legend, and indicate the representative numbers on the images.

The method of quantification has been described in more detail in the methods section (lines 693ff) and the representative numbers have been indicated on the images, as suggested by the reviewer. The legend has been adapted accordingly (lines947ff).

2. In the text, for figures such as Figure 2D, the authors only mention that isolate 101 induces less damage. It would be nice to clarify that this is specifically measured by LDH release, and what this specifically means.

The description of damage induction by 101 was specified in the text, as recommended (line 162ff).

3. For Figure 4D, the authors present their transcriptomic analyses as a subset of differentially regulated genes between isolate 101 and SC5314. It would be informative to show global regulation – what percentage of genes are differentially regulated? Additionally, the text describing this figure seems to attempt to fit all of the presented data to the authors’ hypotheses. Are there any differences that are surprising?

The RNAseq data were analysed in an unbiased way, as described in detail in the methods section. Supplementary Figure S3 displays all enriched GO terms (adjusted p-value < 0.05) with an adjusted p-value < 0.05 on Biological Process and Molecular Function associated with differentially regulated genes between isolates 101 and SC5314. Figure 4B-C represents a summary of those results. The heat map in Figure 4D highlights some of the most strongly regulated genes in gene categories related to the enriched GO terms. The differentially expressed genes linked to filamentation and virulence were not surprising, given the phenotypic data that were already available. In contrast, the metabolic signature of isolate 101 was not anticipated. We were also surprised to find the metabolic profile to be less variable over time than the virulence profile. We have tried to highlight this in the text more explicitly (lines 254 and 257). In response to the reviewer’s valuable suggestion, we’ve added a **new Supplementary Figure S2E** that displays the number of globally regulate genes between SC5314 and isolate 101 and between the different conditions tested. The text in the results section (lines 216ff) and in the supplementary legends has been adapted accordingly.

4. In Figure 6, the authors show that NRG1 deletion results in increased hyphal length. It would be interesting to see whether this deletion restores hyphal length to that of SC5314.

Differences in filamentation length between 101 and SC5314 are shown in Figure 1G. Although the absolute value of the mean filament length of isolate 101 can vary slightly from one experiment to another, comparison of the data in Figure 6C and 1G show clearly that deletion of *NRG1* in isolate 101 does not restore hyphal length to that of SC5314. This also becomes clear when looking at the right-hand plots, where fungal elements were grouped according to their length and shown as the proportion of fungal elements in each group.

5. Figure 6I is not referred to in the text.

We apologize for this error. The reference to the Figure panels 6I-J has now been added in the results section (line 362).
